# Body Composition and Comparison of Diet Quality Using the Healthy Eating Index (2015) and Diet Quality Index-International in a Group of Organic and Conventional Fruit Growers—A Pilot Study

**DOI:** 10.3390/nu18101513

**Published:** 2026-05-09

**Authors:** Hubert Dobrowolski, Bartosz Szumigaj, Dariusz Włodarek, Renata Kazimierczak, Justyna Obidzińska, Ewa Rembiałkowska

**Affiliations:** 1 School of Medical & Health Sciences, VIZJA University, Okopowa 59 Street, 01-043 Warsaw, Poland; h.dobrowolski@vizja.pl; 2 Institute of Human Nutrition Sciences, Department of Functional and Organic Food, Warsaw University of Life Sciences (SGGW), Nowoursynowska 159c Street, 02-776 Warsaw, Polandrenata_kazimierczak@sggw.edu.pl (R.K.);; 3 Institute of Human Nutrition Sciences, Department of Dietetics, Warsaw University of Life Sciences (SGGW), Nowoursynowska 159c Street, 02-776 Warsaw, Poland; dariusz_wlodarek@sggw.edu.pl

**Keywords:** organic food, fruit growers, diet quality, organic consumption, nutritional knowledge, diet quality indices, body composition

## Abstract

**Background/Objectives**: Diet is an essential factor influencing health and the preventive management of some diseases. There is little research to date on the diet of organic food producers, particularly organic ones. The present study aimed to investigate the diet quality of organic and conventional fruit growers. **Methods**: Fifty-three fruit growers, including 28 organic and 25 conventional, took part in the study. Body weight and height were measured. Body composition was estimated using the BIA method. Information on dietary intake was collected using a 3-day dietary record. Diet quality scores were calculated using the Healthy Eating Index-2015 (HEI-2015) and the Diet Quality Index International (DQI-I). **Results**: The diet quality of the orchardists was low (31.7 ± 9.3 points on the HEI scale and 54.3 ± 7.5 points on the DQI-I scale). Organic fruit growers scored significantly higher on both scales, compared to conventional fruit growers (*p* < 0.001 and *p* = 0.002 for HEI and DQI-I, respectively) and had a lower percentage of BF and a higher percentage of FFM and TBW in their bodies (*p* = 0.013, *p* = 0.049 and *p* = 0.049, respectively). Consumers of organic products had better diet quality scores, and diet quality increased as the percentage of organic food consumption increased. Both diet quality and the percentage of organic food consumed were associated with participants’ self-assessed nutritional knowledge. **Conclusions**: Diet quality was associated with the type of agricultural practice and with organic food consumption. As a pilot cross-sectional study based partly on self-reported measures, the observed differences should be interpreted cautiously. They may also partly reflect broader lifestyle-related factors. Further research on similar groups is needed to confirm these relationships, preferably with an assessment of respondents’ nutritional knowledge.

## 1. Introduction

Diet and nutritional composition are crucial in the prevention and, in many cases, in the treatment of several diseases [1]. However, due to the large number of nutrients and antinutrients in foods, as well as interactions that may occur between nutrients from different sources, it is important to consider diet more broadly, in addition to the intake levels of individual nutrients [1]. To this end, it is useful to assess the overall quality of the diet and dietary patterns rather than focusing solely on individual dietary components. Dietary quality indicators are tools used to assess intake of individual dietary components, food products, and food groups, or combinations of these factors [2]. Therefore, these indicators are often used to assess the compliance of a diet with dietary recommendations, to track healthy dietary patterns or to monitor the prevalence of chronic diseases in specific population groups [3,4].

Several factors influence diet, such as financial situation, health status, occupation or household management. Only a few studies reported that specific dietary patterns will also characterise farmers and fruit growers, as well as their co-workers and families [5,6,7,8,9]. Specific diets, as well as the quantity and frequency of food consumption, including individual food groups, will distinguish these individuals. Previous Polish studies have shown that the diets of these groups are based on flour, bread, pasta [5,6], fruit and vegetables, eggs, milk and meat [5], but beef consumption is lower than in other social groups [7]. Other studies have shown that people involved in food production are more likely to consume products they produce themselves [8].

Due to the nature of the work of farmers and fruit growers, the frequency of meals differs from that of the general population. Fruit growers often spend whole days in the orchards, with very little time for meals, which affects both their poor eating habits and the quality of their diet. Although there are general comparisons of the dietary quality of rural and urban populations [9,10,11,12], no specific studies on the dietary quality of farmers and fruit growers have been found to date. A special method of farming and fruit growing is organic farming [13,14]. Some studies indicate that farmers often consume ingredients and products they produce themselves [8]. It can therefore be assumed that organic fruit growers are likely to have grown their own fruit. At the same time, as previous studies indicate, consumers of organic products have a more balanced diet that aligns more closely with nutritional recommendations [15,16]. Therefore, it can be hypothesised that organic fruit growers have a diet closer to the recommendations of nutritionists than conventional fruit growers. An additional hypothesis is that there is probably a positive correlation between organic food consumption and diet quality. This occupational group is of particular interest because fruit growers may differ from the general population in seasonal workload, rural food access, reliance on self-produced foods, and exposure to production-related values and practices. In this context, comparing organic and conventional fruit growers may help to identify whether different dietary patterns and body-composition profiles accompany different farming systems; however, given the pilot and cross-sectional design, such comparisons should be understood as exploratory rather than explanatory.

To date, few studies have analysed the diet quality of organic and conventional fruit growers, which is why the present study was considered highly necessary.

The composition of the diet, and thus its quality, as reflected in appropriate indicators, may be reflected in changes in body composition. This relationship has been demonstrated in some [17,18,19] but not all [20] studies. The way orchard farming is conducted can also affect body composition. To our knowledge, however, no studies have been conducted in Poland on differences in body composition between organic and conventional fruit growers, and foreign studies are very few.

The presented research was conducted as part of the CO-FRESH project (CO-creating sustainable and competitive FRuits and vEgetableS’ value cHains in Europe). The main objective of the CO-FRESH project was to develop innovative systemic approaches to value chains in the agri-food sector, increasing their scale at the European level. In Poland, the pilot unit was the association of organic fruit producers, EKOOWOC, which brought together 20 fruit growers in the Mazowieckie Province.

Given the gaps in the literature and the enormous impact of diet on health, this pilot study aimed to compare body composition and diet quality between organic and conventional fruit growers. More specifically, the study was designed to explore whether orchard management type and reported organic food consumption were associated with diet quality and body-composition parameters in this occupationally relatively homogeneous group.

## 2. Materials and Methods

### 2.1. Study Group

A total of 53 fruit growers participated in the study. Twenty-eight participants were from an organic orchard (16 orchardists and 12 partners), and 25 participants were from a conventional orchard (15 orchardists and 10 partners). The partners of the fruit growers share similar lifestyles and physical activity due to their assistance with the fruit growers’ daily chores, as well as a similar worldview on environmental issues and a similar diet, resulting from shared family meals. Recruitment was done with the help of organic fruit growers’ associations. From the selected fruit growers who agreed to participate in the study, conventional fruit growers who lived in the nearest neighbourhood were selected. This ensured similar product availability in local shops and farms, as well as similar environmental conditions. Participants under 20 or over 65 years of age were excluded from the study (a total of 4 participants) due to differences in physical activity, lifestyle, and nutritional needs, which would have altered the results. The gender distribution, financial situation, and level of education are presented in Table 1.

Approval for the study was obtained from the Rector’s Committee on Ethics in Human Research at SGGW (decision no. 7/RKE/2023/U of 30 January 2023). All participants were informed about the study, the measurements to be taken, the data to be collected and the use of the information collected during the study. Written consent was obtained from each individual to participate in the study and in any research procedures. All questions and concerns of the study participants were answered in detail by the research team carrying out the measurements.

### 2.2. Data Collection

The research was carried out in November–February (2023–2024), a period during which no intensive work is done in the orchards, and fruit growers are more accessible as respondents. This period is characterised by reduced agricultural activity by orchardists. There is limited exertional activity and, consequently, a lower physical activity rate. The reduced orchard activity among participants in the study allowed them to devote more time to their diet diary, which may have translated into higher-quality data. All measurements and interviews were carried out directly at the fruit growers’ homes. This provided stable measurement conditions and comfort for the respondent, enabling the collection of higher-quality data. All measurements and interviews were conducted by the study’s co-authors, who are trained researchers with specialised backgrounds in food and nutrition.

#### 2.2.1. Anthropometric Measurements

The study participants had their height, body mass and body composition measured twice. If the measurement results deviated significantly, another (third) measurement was taken. The two measurements most similar to each other were then used for further analysis. An arithmetic mean was drawn from the two measurements. Body mass and body composition were measured using a Tanita BF-350 device (Tanita Corp., Tokyo, Japan). For the measurement, participants removed their outer clothing and any metal jewellery. Body height was measured using a SECA 213 stadiometer (Seca GmbH & Co. KG, Hamburg, Germany). Measurements were taken without shoes and socks. The head arrangement corresponded to the Frankfurt plane position.

#### 2.2.2. Dietary Data

The diets of the study participants were obtained using the 3-day dietary record method. This traditional method is usually used to assess dietary intake [21] and as a reference standard to document current intake [22]. The recording of food consumed took place over 3 days: 2 working days and 1 day off. Participants were carefully instructed on how to complete the questionnaire, the need to accurately report the composition and recipe of the food and all products consumed, and the need to record the amount of food consumed immediately after consumption.

The data obtained were analysed using Dieta 6.0 software (NIZP-PZH, Warsaw, Poland). This provided data on the energy value of the diet, the amounts of saturated, monounsaturated, and polyunsaturated fatty acids, the content of sodium, added sugars, cholesterol, vitamins, and minerals, and the number of macronutrients consumed. These values were then used to determine diet quality indices. The software uses the most up-to-date tables of food composition and nutritional value in Poland [23].

#### 2.2.3. Questionnaire

Physical activity was assessed using the Johansson and Westerterp questionnaire [24]. It is a simple, useful questionnaire in which respondents specify the intensity of physical activity at work and during leisure time. The answers to these questions allow for the estimation of the physical activity level (PAL). This questionnaire was previously validated using the doubly labelled water method [24]. In addition, questions on organic food consumption and self-assessment of nutritional knowledge and diet were used. Respondents were asked to self-assess on a 5-degree Likert scale (possible answers: bad; rather bad; neither good nor bad; rather good; good) their knowledge of food and nutrition, as well as their diet. Regarding the questions on organic food, respondents were asked whether the participant consumes organic food (possible answers: yes; no), how long they have been consuming organic food (possible answers: less than 1 year; 1–3 years; more than 3 years) and in what percentage organic food is consumed (possible answers: less than 20%; 20–40%; 41–60%; 61–80%; more than 80%). For the binary analyses, participants who answered “yes” to the habitual organic food consumption question were classified as organic food consumers, whereas those who answered “no” were classified as non-consumers.

### 2.3. Data Analysis

#### 2.3.1. Diet Quality Assessment

The DQI-I [25] and the HEI-2015 [26] indices were used as indicators of dietary quality. The DQI-I is based on scoring 17 components divided into 4 subcategories: variety (containing overall food group variety and within-group variety for protein source), ade-quacy (containing including an assessment of the intake of the number of servings of veg-etables, fruit and cereal products, a quantitative assessment of fibre intake and an assess-ment of the coverage of protein, iron, calcium and vitamin C needs), moderation (includ-ing an evaluation of intake of total fat, saturated fat, cholesterol, sodium and empty calorie foods) and overall balance (including an assessment of the macronutrient ratio and the proportion of fatty acid intake PUFA:MUFA:SFA). Values for these indicators are assigned points based on the category, ranging from 0 to 15. A total of 20 points can be obtained in the variety category, 40 points in the adequacy category, 30 points in the moderation category and 10 points in the overall balance category. The number of points obtained indicates the quality of the diet, with higher scores indicating higher quality. A final score of 0–100 points could therefore be achieved. To calculate the scores, we used the authors’ original guidelines [25]. The HEI-2015, in turn, scores on a scale of 0–5 a consumption of fruit and juice, vegetables (including greens, beans, peas), protein products, seafood and vegetable protein, and on a scale of 0–10 whole grains, dairy, fatty acid ratio, refined grains, sodium, added sugars and saturated fats. For each of these features, the index authors specify the minimum and maximum number of points. Intermediate points are calculated on a ratio basis. The index can be scored from 0 to 100, with higher values indicating a better overall diet quality. The authors’ indications were used as cut-off points: 90–100, grade A; 80–89, grade B; 70–79, grade C; 60–69, grade D; 0–59, grade E. The HEI-2015 also includes 2 subcategories: adequacy components (total fruits, whole fruits, total vegetables, greens and beans, whole grains, milk and dairy, total protein foods, seafood and plant proteins, fatty acids) and moderation components (saturated fats, refined grains, sodium, added sugars). All the determinants present in both the HEI-2015 and the DQI-I were obtained after the dietary record was collected and analysed using the appropriate software (in this case, Diet 6.0). Ratings were made using individual scoring scales for each day and for the average across all 3 days. The binary analyses of organic food consumption were therefore based on this self-reported yes/no classification and should be interpreted as reflecting habitual declared consumption.

#### 2.3.2. Statistical Analysis

Statistical analysis was performed using SPSS v. 28.0 (IBM Corp., Armonk, NY, USA), R (4.3.1), and RStudio (4.3.1) (The R Foundation, Vienna, Austria). The normality of the data distribution was estimated using the Shapiro–Wilk test. The comparisons between organic and conventional fruit growers, between the genders of the study participants, and between the fruit growers and their partners were performed using Student’s *t*-test for normal distributions and the U Mann–Whitney test for non-normal distributions. Comparisons between diet quality and self-assessed nutrition knowledge, self-assessed diet, and organic food consumption (in terms of quantity and for how long participants consumed organic food) were made using ANOVA. A χ^2^ test was used to assess the relationship between orchard management type and organic food consumption. Correlations between normally distributed data and quantitative variables were assessed using Pearson’s test. Correlations between variables with distributions deviating from normality and between ordinal variables were tested using Spearman’s test. The study’s defined significance level was set to α = 0.05.

## 3. Results

### 3.1. Anthropometric Data

The anthropometric data for the study group are presented in Table 2. The study group had an average age of 44 ± 8 years (with no statistical differences between subgroups). Mean height and weight were 172 ± 9 cm and 84 ± 16 kg, respectively. The mean BMI in the study group was 28 ± 4 kg/m^2^. The fruit growers had greater height (*p* < 0.001, Student’s *t*-test) and body weight (*p* < 0.001, Student’s *t*-test) than their partners (due to the gender imbalance between the groups, with a majority of partners being women). Also, the mean BMI of orchardists was higher than that of their partners (*p* = 0.017, Student’s *t*-test). The median PAL in the study group was 1.7 (1.4–2.3). Fruit growers had a significantly higher PAL compared to their partners (*p* = 0.037, U Mann–Whitney test). No differences were observed between the organic and conventional fruit growers and their partners in terms of age, weight, height, and BMI (*p* > 0.05, Student’s *t*-test) and PAL (*p* > 0.05, U Mann–Whitney test).

The body composition of the study participants is presented in Table 3. The median percentage of body fat in the study group was 28.5% (14.2–45.4%), while the median percentage of total body water was 52.4% (40.0–62.8%). The median fat-free body mass was 57.8 kg (40.0–80.2 kg). No relationship was found between the conventional and organic groups for the parameters studied (*p* > 0.05, Mann–Whitney U test) when both fruit growers and their life partners were included in the analysis. However, the analysed group of fruit growers showed a significantly higher lean body mass than their partners (*p* = 0.013, Mann–Whitney U test). A significantly lower percentage of body fat (*p* = 0.049, Mann–Whitney U test) and a significantly higher percentage of body water (*p* = 0.049, Mann–Whitney U test) were observed in the group of organic fruit growers compared to the group of fruit growers using conventional farming methods. These differences were not observed when comparing groups of partners by farming method (*p* > 0.05, Mann–Whitney test). No relationships were found between organic food consumption and body composition parameters (*p* > 0.05, Mann–Whitney test).

### 3.2. Overall Diet Quality Results

According to the HEI-2015, the study group scored from 16 to 54 (mean: 31.7 ± 9.3) points. The organic fruit grower group had a statistically significantly higher mean score than the conventional fruit grower group, respectively 37.0 ± 6.7 points vs. 25.8 ± 8.2 points (*p* < 0.001, Student’s *t*-test) (Table 4). There were no statistically significant differences in HEI-2015 scores between the fruit grower and partner groups, or according to gender (*p* > 0.05, Student’s *t*-test).

Using the DQI-I, the score ranges from 33 to 69 (mean: 54.3 ± 7.5) points. The group of organic fruit growers obtained significantly better results (57.1 ± 7.1 points) compared to conventional fruit growers (51.2 ± 6.7 points) (*p* = 0.002, Student’s *t*-test) (Table 2). Also mean score of the DQI-I was significantly higher for women than men (respectively: 56.7 ± 6.6 and 52.4 ± 7.8; *p* = 0.016, Student’s *t*-test) and for partners than fruit growers (respectively: 57.1 ± 6.7 and 52.4 ± 7.5; *p* = 0.012, Student’s *t*-test).

### 3.3. Detailed Structure of Diet Quality Indicators

**Figure 1:**
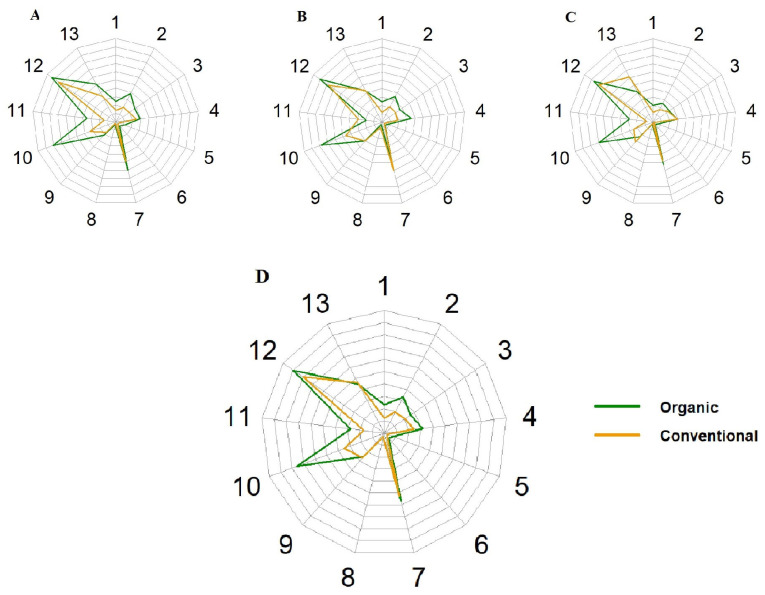
Structure of scores in individual HEI-2015 classification domains. (**A**) Point allocated for Day 1; (**B**) Point allocated for Day 2; (**C**) Point allocated for Day 3; (**D**) Average scores for all 3 days. 1—Total Fruits; 2—Whole Fruits; 3—Total Vegetables; 4—Greens/Beans; 5—Whole Grains; 6—Dairy; 7—Total Protein Foods; 8—Seafood/Plant Proteins; 9—Fatty Acids; 10—Refined Grains; 11—Sodium; 12—Added Sugars; 13—Saturated Fats.

The structure of the scores obtained in each category (HEI-2015) is shown in the diagram (Figure 1). Organic orchardists scored significantly higher than conventional orchardists in domains: total fruits, whole fruits, refined grains and whole grains (for all *p* < 0.01, U Mann–Whitney test). Also, they had higher scores in the following domains: added sugar, sodium, total protein foods, dairy, greens and beans, and total vegetables, but the differences were not statistically significant.

**Figure 2:**
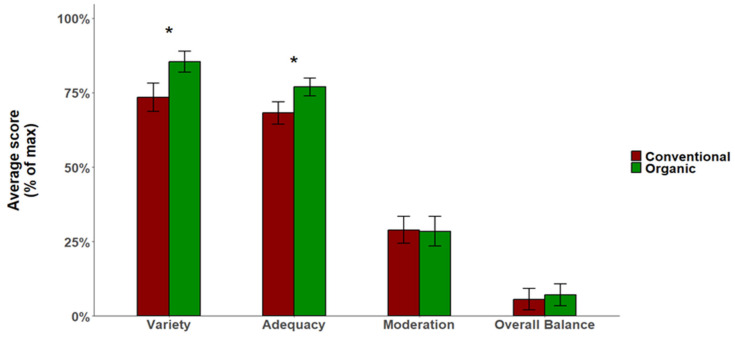
The scores obtained in the 4 main categories of the DQI-I indicator. * *p* < 0.001, U Mann–Whitney test.

When analyzing the structure of responses with the DQI-I, the surveyed group median of obtained points in 4 main categories were: the variety (15.9 points out of 20 points maximum; 79.4%), the adequacy (29 points out of 40 points maximum; 72.6%), the moderation (8.6 points out of 30 points maximum; 28.7%) and the overall balance (0.6 points out of 10 points maximum; 6.37%). The group of organic fruit growers scored significantly higher in terms of variety (*p* < 0.001, U Mann–Whitney test) and adequacy (*p* < 0.001, U Mann–Whitney test) (Figure 2).

**Figure 3:**
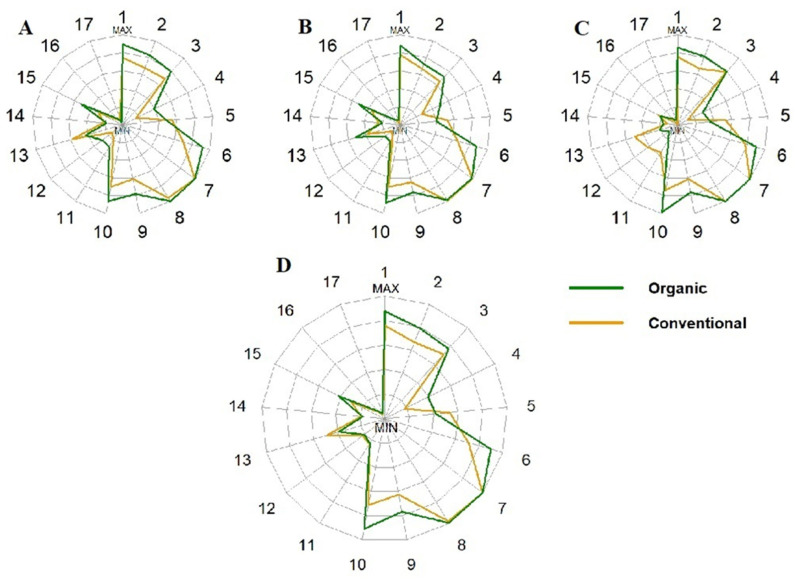
Structure of scores in individual DQI-I classification domains. (**A**) Point allocated for Day 1; (**B**) Point allocated for Day 2; (**C**) Point allocated for Day 3; (**D**) Average scores for all 3 days. 1—Overall variety; 2—Variety for protein; 3—Vegetables; 4—Fruits; 5—Grains; 6—Fiber; 7—Protein; 8—Iron; 9—Calcium; 10—Vitamin C; 11—Total fat; 12—Saturated fat; 13—Cholesterol; 14—Sodium; 15—Empty calorie foods; 16—Macronutrients ratio; 17—Fatty acid ratio.

Analysing the components of the index in more detail, it was noted that organic fruit growers scored more through the following domains: overall food group variety, within-group variety for protein source, fruit group, fibre, Vitamin C (medians, *p* < 0.05, U Mann–Whitney test), grain group, calcium (means, *p* < 0.05, Student’s *t*-test) than conventional fruit growers. In other domains, no statistically significant differences were observed (*p* > 0.05, U Mann–Whitney test). The scheme (Figure 3) shows the distribution of scores in each DQI-I category.

### 3.4. Diet Quality and Self-Assessment of Nutritional Knowledge and Eating Practices

There was a relationship between self-assessed nutritional knowledge and HEI-2015 scores (*p* = 0.014, ANOVA). A post hoc analysis showed that those rating their nutritional knowledge as very good scored higher than those rating it as neither good nor bad (*p* = 0.026, Scheffe test). A relationship was also found between self-assessed nutritional knowledge and DQI-I scores (*p* = 0.005, ANOVA). Post hoc analysis showed that those rating their knowledge as either very good (*p* = 0.017, Scheffe test) or good (*p* = 0.022, Scheffe test) scored better than those rating their knowledge as neither good nor bad. These findings refer to self-assessed rather than objectively measured nutritional knowledge.

However, no differences were found between self-assessed nutrition practices and the HEI and DQI-I indices (*p* > 0.05, ANOVA).

A correlation was found between the self-assessment of nutritional knowledge and the number of HEI-2015 (*p* < 0.001, rho = 0.431, Spearman test) and DQI-I (*p* = 003, rho = 0.403, Spearman test) points scored.

### 3.5. Diet Quality and the Consumption of Organic Food

Organic food consumers had better diet quality scores using the HEI-2015 indices (34.71 ± 8.4 points) than non-organic food consumers (24.12 ± 6.9 points). This relationship was statistically significant (*p* < 0.001, Student’s *t*-test). All respondents who consumed organic food reported doing so for more than 1 year. The HEI scores for organic food consumers for 1–3 years (HEI score: 36 ± 1) and for over 3 years (HEI score: 34 ± 8) were significantly higher than for not consuming such food (HEI score: 24 ± 7) (respectively: *p* = 0.004 and *p* = 0.001; Scheffe test). There was also a relationship between the declared amount of organic food consumed and HEI-2015 results (*p* = 0.002, ANOVA). Those declaring to consume between 20 and 40% organic food in their dietary ration had significantly higher HEI-2015 scores (36 ± 8 points) than those who did not consume organic food at all (24 ± 7 points) (*p* = 0.011, Scheffe test). In these comparisons, the term “organic food consumers” refers to participants who reported habitual organic food consumption in the binary questionnaire item.

The DQI-I score for the group of organic food consumers was 54.98 ± 7.97 points, and for the group not consuming such food was 52.7 ± 6.1 points, but these differences were not statistically significant (*p* < 0.05, Student’s *t*-test). Also, no significant differences were found according to the percentage of organic food consumed and how long organic food had been consumed (*p* < 0.05, ANOVA).

We found a significant relationship between orchard management type and organic food consumption (*p* < 0.001, χ^2^ = 17.891). Those managing an organic orchard consume more organic food than those managing a conventional orchard.

**Figure 4:**
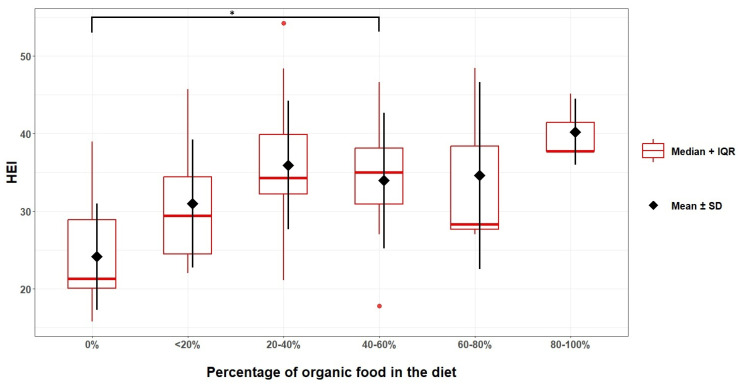
Relationship between the scores obtained with the HEI-2015 and the percentage of organic food consumed. * *p* = 0.011, Scheffe test.

**Figure 5:**
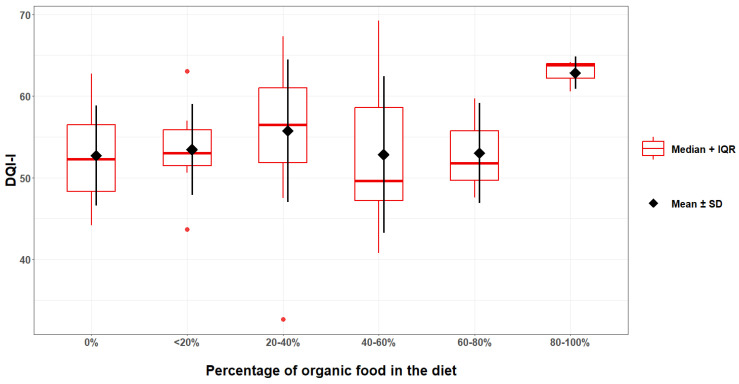
Relationship between scores obtained with the DQI-I and the percentage of organic food consumed.

Figure 4 and Figure 5 show the relationship between the percentage of organic food consumed and the scores obtained according to the HEI-2015 and DQI-I indices (Figure 4 and Figure 5).

### 3.6. Correlations

**Figure 6:**
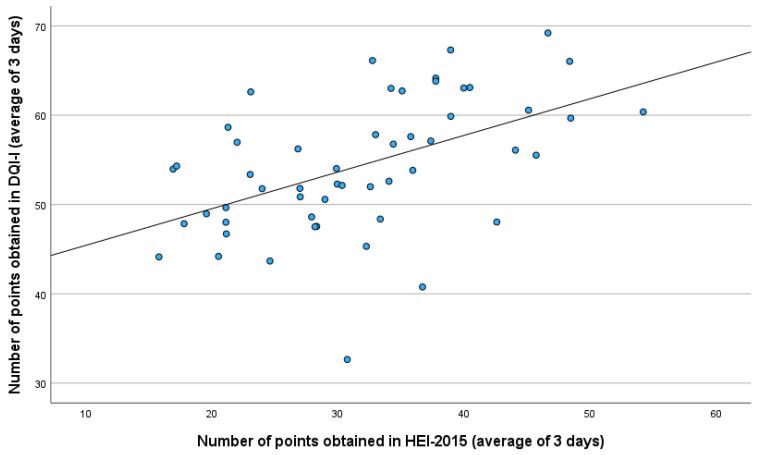
Relationship between scores obtained with the DQI-I and the HEI-2015 indices.

There was no correlation between the age of the study participant and the values indicated by the HEI-2015 (*p* = 0.362, r = 0.125, Pearson test) and DQI-I (*p* = 0.669, r = −0.060, Pearson test). Also, there was no correlation between BMI and HEI-2015 results (*p* = 0.936, r = −0.011, Pearson test). However, there was a correlation between BMI and DQI-I scores. (*p* = 0.015, r = −0.331, Pearson test). We found a correlation between the results of the DQI-I and HEI-2015 indicators (*p* < 0.001, r = 0.506, Pearson test) (Figure 6). A correlation was also found between self-assessed nutritional knowledge and the percentage of organic food consumed (*p* < 0.001, rho = 0.473, Spearman test). No correlations were found between the HEI-2015 and DQI-I indices and body composition parameters in the study group (*p* > 0.05, Spearman test). However, when only the group of fruit growers was included in the analysis and the group of partners was excluded, a negative correlation was found between the HEI-2015 index and the percentage of body fat (*p* = 0.036, rho = −0.375, Spearman test), percentage of total body water (*p* = 0.038, rho = 0.375, Spearman test) and fat-free mass (*p* < 0.001, rho = 0.572, Spearman test), and between the DQI-I index and the percentage of body fat (*p* < 0.001, rho = −0.712, Spearman test), percentage of total body water (*p* < 0.001, rho = 0.712, Spearman test) and fat-free mass (*p* = 0.046, rho = 0.361, Spearman test). No correlation was found between PAL and BMI (*p* = 0.358, rho = 0.129, Spearman test), PAL and HEI-2015 and DQI-I indices (*p* > 0.05, Spearman test), or between PAL and body composition parameters (*p* > 0.05, Spearman test).

## 4. Discussion

The results presented here are the first to provide insight into the qualitative aspects of fruit growers’ diets. It is also the first study to compare the diet quality aspects of organic and conventional producers. The relatively small sample size should be interpreted in the context of the target population, as the total number of organic farms in Poland was 23,155 in 2024 (Agricultural and Food Quality Inspection, Poland, 2024, https://www.gov.pl/web/ijhars/dane-o-rolnictwie-ekologicznym; accessed on 22 December 2025), of which only a proportion are horticultural farms. The relevance of studying fruit growers specifically lies in the fact that their work organisation, family participation in farm duties, seasonal rhythms, and access to self-produced foods may shape eating patterns differently from those of the general population. At the same time, organic and conventional production systems may be associated with different food-related attitudes and behaviours; nevertheless, the present study cannot determine whether such factors are causal.

Few studies have addressed diet quality among consumers of organic and conventional foods. The present study, therefore, makes a valuable contribution to knowledge of diet quality and, thus, disease risk among organic and non-organic farmers and orchardists. This study presents the relationships between the HEI-2015 and DQI-I indicators and the way the orchard farm is run, as well as the consumption of organic food. Although our study included a relatively small sample, the findings suggest that organic orchardists have more favourable dietary patterns than conventional orchardists. Accordingly, the present findings should be interpreted as an exploratory signal from a narrowly defined occupational group rather than as evidence directly transferable to the broader population.

### 4.1. Interpretation of Diet-Quality Differences

The 2015 Healthy Eating Index (HEI) is used to assess compliance with the American dietary recommendations [27]. It is an accurate and reliable method for assessing diet quality in relation to these guidelines [28]. Several studies have linked higher HEI scores with lower all-cause mortality and risk of type 2 diabetes, cardiovascular disease, and cancer [29,30,31,32,33]. Diet Quality Index—International has built on its predecessors—DQI [34] and its successors DQI-R [35]. The DQI-I in its design emphasises four major aspects, including a high-quality, healthy diet, i.e., variety, adequacy, moderation and overall balance [36] and is internationally applicable [25]. As several studies indicate, both the HEI and DQI are valid measures of overall diet quality based on food group consumption, nutrient intake associated with chronic disease, and diet variety [37].

Regardless of the indicator used, the diet quality in the study group was unsatisfactory. In the HEI-2015, no participant scored 59 points, indicating that all participants received the lowest diet quality category. For the DQI-I, the average score was 54.3 ± 7.5, indicating a score hovering around the midpoint of the scale. It is also worth noting that more than half (68%) of the study participants exceeded the 50-point threshold. However, there are no specific cut-off points, unlike the HEI-2015. However, given the scores’ oscillation in the middle of the scale and the HEI classification, the diet’s quality should be considered average at best. In their study, Tur et al. (2005) reported that a DQI-I score of 43% indicated a poor-quality diet [37]. Mariscal-Arcas et al. (2010), on the other hand, in their work with schoolchildren, pointed out that, although a score of around 58% on the DQI-I scale was indicative of a poor quality diet, given the differences in the Mediterranean region (higher intake of fats, especially MUFAs), this score did not necessarily indicate poor diet quality [38]. For the Polish population, however, the dietary pattern is far from the Mediterranean model. Low scores on diet quality indicators can increase the risk of health consequences [29,30,31,32,33,39,40,41,42,43]. Similarly, other studies evaluating dietary patterns in rural populations (using DQI) have found that overall diet quality is rather low [44,45,46]. In other studies employing the HEI questionnaire, both its more recent and earlier versions yielded higher scores; nevertheless, overall diet quality tended to be classified as poor or very poor [47,48]. Thus, the study shows a comparable trend towards poor diet quality in the rural population. However, the group of organic fruit growers had significantly more points than the group of conventional fruit growers and was much closer to achieving a better diet quality score. This may suggest that the degree of exposure to negative health consequences from poor diet quality is also lower in this group, a finding that certainly warrants further research.

If we take a closer look at the difference in scores between organic and conventional fruit growers in our study, both the HEI-2015 and the DQI-I indicate a more favourable consumption of whole grain products (whole grain group in HEI-2015; grain group and fibre in DQI-I) and fruits (whole fruits and total fruits in HEI-2015; fruit group, vitamin C and fibre in DQI-I). Adequate consumption of whole grains, fruits and fiber is recommended by numerous positions, including the recommendations of the World Health Organization [49], Dietary Guidelines for Americans [50], Polish Healthy Eating recommendations (by National Institute of Public Health) [51], American Heart Association [52], International Agency for Research on Cancer [53], and many others, due to their health-promoting properties and their roles in the prevention of many non-communicable diseases. In addition, differences in DQI-I between the organic and conventional fruit grower groups were observed for overall variety and variety of protein sources, suggesting a greater dietary diversity among organic fruit growers. However, variety is not separately scored in the HEI-2015 index; therefore, no observed relationships are observed in this indicator. The same is true for the calcium component, which is not specified in the HEI-2015 but is scored based on the assessment of the individual components that contain calcium. Many studies to date have shown a relationship between the share of organic food in the diet and the degree of compliance with dietary recommendations. This is indicated by studies conducted, among others, in France [54], Denmark [55], Poland [16], and Germany [56].

Of the four categories, organic fruit growers also scored significantly higher on variety, including the above-mentioned components of overall variety and variety for protein source, and adequacy. They also scored higher on overall balance, but the differences were not statistically significant. For the fourth category, moderation, practically no differences were observed (Figure 2), indicating a very positive trend. As shown in a large study on the Chinese population, higher scores on exactly these 3 categories were associated with a reduction in all-cause mortality, and 20.1%, 31.3% and 13.9% of deaths could be avoided by improving these indicators (for variety, adequacy and overall balance, respectively) [57]. This therefore indicates that organic orchardists had a slightly better-quality diet than conventional orchardists, which, if a more extensive study confirmed our results, could yield numerous health benefits for them.

### 4.2. Self-Assessed Nutritional Knowledge and Organic Food Consumption

The better diet quality observed among organic fruit growers in our study may have been due to greater nutritional knowledge. As the results indicate, the organic fruit grower group rated their nutritional knowledge better than the conventional fruit grower group (*p* < 0.001, U Mann–Whitney test). It should be noted, however, that self-assessed nutritional knowledge does not necessarily translate into actual knowledge. As Jeżewska-Zychowicz and Plichta (2022) noted, declarative knowledge does not necessarily translate into skills or the ability to choose healthier foods [58]. It is worth noting, however, that in the aforementioned study, the authors assessed nutritional knowledge using a test rather than asking respondents to self-assess. In our case, however, the declarative nutritional knowledge may have had an impact on the quality of the diet, as it can be observed that the groups of people with higher declared knowledge had a better quality of the diet, both shown by the HEI-2015 and the DQI-I (respectively: *p* = 0.014 and *p* = 0.005, ANOVA). This is also indicated by the observed correlation between the level of nutritional knowledge and the results obtained through the HEI-2015 (*p* < 0.001) and DQI-I (*p* = 0.003). This may therefore suggest that the organic fruit growers were indeed more interested in proper nutrition and dietary recommendations than the conventional fruit growers, and thus assessed their knowledge of nutrition more thoroughly. However, the knowledge obtained in this way was often insufficient to enable them to balance their diets and adopt healthy eating patterns. The lack of actual testing of nutritional knowledge among the respondents, however, makes it impossible to conclusively verify whether better indicators of diet quality may be related to better nutritional knowledge in the study group. Importantly, this construct was self-assessed and may also reflect broader health consciousness, motivation, and engagement with food-related information rather than solely objective knowledge. For this reason, the observed associations should not be overinterpreted as proving a direct effect of nutritional knowledge on diet quality.

The question of whether participants in this study who consume organic food have better diet quality than non-organic consumers needs clarification. Indeed, it has been observed that orchard management (organic vs. conventional) can influence diet quality parameters and their alignment with dietary recommendations. However, running an organic orchard does not guarantee that an organic orchardist consumes organic food, and a conventional orchardist does not. Even if an organic orchardist were to consume organic food, this does not necessarily mean they consume a higher percentage of their total ration than conventional orchardists. While this is obviously an assumption, it is not necessarily correct. In our work, we detected a relationship between orchard management type and organic food consumption, but without any indication of direction or quantitative comparison. Nevertheless, consumers of organic food scored significantly better on the HEI-2015 than those who did not consume organic food. Also, using the DQI-I, consumers of organic food obtained better results, but no significant relationship was found. Similar observations can be seen for the period since the study participants began consuming organic food. Study participants who had been consuming organic food for more than one year (1–3 years; more than 3 years) scored significantly better than participants who did not consume organic food. Also, for the DQI-I, a similar relationship is observed, though it is not statistically significant. Finally, as the proportion of organic food in the dietary ration increases, a clear trend can be observed (Ryc. 4; Ryc. 5), indicating an improvement in diet quality indicators, using both the HEI-2015 and the DQI-I indices. The observed correlations may be due to self-assessed nutritional knowledge. Indeed, it has been shown that as declarative nutritional knowledge increases, the consumption of organic food also increases (*p* < 0.001). This, therefore, indicates that, along with gaining knowledge about food and nutrition, participants encountered information on the health-promoting properties of organic food and thus decided to increase their consumption. Similar results were observed in a study by Pieniak et al. (2010) [59], which found that consumption of organic vegetables was associated with subjective knowledge. Nevertheless, the authors of this study observed that objective knowledge was only indirectly related to the consumption of organic vegetables [59]. It must be noted that our questions were about general subjective nutritional knowledge, whereas the authors asked about their assessment of their knowledge of organic food. The opposite conclusion was reached in the paper by Teng & Wang (2015), which found that the effect of perceived organic knowledge on consumer attitudes was insignificant, suggesting that increased perceived knowledge cannot create positive attitudes towards organic food [60]. They also surveyed participants’ knowledge of organic food, which may make comparisons with the present study’s results difficult. Interesting results were obtained in a study by Padilla Bravo et al. (2013), which showed that participants who used multiple sources of food information had more positive feelings towards organic food and were more likely to buy it [61]. Naturally, the use of multiple sources of information does not necessarily affect nutritional knowledge, but it certainly affects the perception of the knowledge held, as observed in this study. However, as mentioned, the participants’ actual nutritional knowledge was not tested in this study. Therefore, it is not possible to clearly assess whether nutritional knowledge had a simultaneous effect on increasing organic food consumption and improving diet quality indicators. In this study, it was observed that subjectively perceived and declared nutritional knowledge was strongly related to the percentage of organic food consumed and to diet quality. The observed differences may also be partly explained by residual confounding from education, perceived financial situation, general health consciousness, or other lifestyle-related factors that were not captured in sufficient detail for multivariable adjustment in this pilot analysis.

### 4.3. Body Composition Findings

It is worth noting that in our study, we did not find a significant difference in BMI between organic and conventional fruit growers. The same applies to comparisons of the BMI of the life partners of organic and conventional orchardists. The results we obtained are in contradiction with those obtained by other authors. In a large-scale study, Baudry et al. (2018) found that organic food consumers had significantly lower BMIs than non-organic food consumers [62]. Also, similar results were reported by Gosling et al. (2021), who noted a clear trend between organic food consumption and BMI [63]. However, these authors investigated the relationship between BMI and organic food consumption. In the present study, although we detected an association between orchard management type and organic food consumption, this does not necessarily imply that organic orchardists actually consumed more organic food; thus, the association was not necessarily observed. Another, more likely, reason for the lack of visibility of such a relationship is the low number of respondents, which was insufficient to achieve adequate statistical power.

As with BMI, no differences in body composition were observed between the conventional and organic groups when comparing fruit growers with their life partners. However, when the life partners of the studied fruit growers were excluded from the analysis, differences emerged between the organic and conventional fruit grower groups in percentage body fat and total body water. A similar analysis was conducted in Thailand by Kongtip et al. (2018) [64]. Although a difference between the groups was found in the BMI index, no difference was observed in the percentage of body fat [64]. There are many potential explanations for this phenomenon. One of them is the use of pesticides by conventional fruit growers, which increases the risk of obesity [65,66]. This would also explain the lack of similar observations among fruit growers’ partners, who have less contact with pesticides than fruit growers do. However, this study did not account for the use of plant protection products, so it is difficult to say whether this affected the occurrence of the phenomenon. Another potential explanation could be the more frequent use of machines by conventional rather than organic fruit growers, as observed in studies [67] reduced use of machines may result in more intense physical work, thereby increasing the level of physical activity and leading to a more optimal body composition—lower body fat content. Lastly, body composition may depend on diet, both in terms of the quantity of nutrients consumed and their quality. In this study, a correlation was indeed observed between diet quality indicators, both HEI-2015 and DQI-I, and individual body composition parameters. However, this is only an observation of certain relationships, and the study’s cross-sectional design does not allow for a clear determination of causality. The results indicate the need for further research to clarify the relationships described.

Finally, the results obtained with the HEI-2015 were strongly correlated with those obtained through the DQI-I. Although the HEI-2015 is used to assess dietary compliance with US dietary recommendations, both indices examine the qualitative aspect of the diet. The US dietary recommendations, in fact, directly address aspects of dietary healthiness, and their application would certainly improve their quality. In addition, many components of the two indicators are identical, such as the consumption of whole-grain products and fruit and vegetables. A correlation of the two indicators was therefore to be expected. In some places, however, one indicator is more sensitive than the other. The HEI-2015 found a correlation between organic food intake and diet quality, whereas in the DQI-I, the correlation was not statistically significant; only a trend was observed. On the other hand, the DQI-I correlated with BMI, with higher BMI associated with lower DQI-I scores. This indicator is therefore more sensitive to the overweight aspect than the HEI-2015, for which no such correlation was observed. It seems advisable to use multiple indices together to avoid overlooking important qualitative aspects of diet and its determinants.

### 4.4. Strengths and Limitations

Our study has both strengths and limitations. Undoubtedly, a strength is that the study was carried out with a group of fruit growers, which no one has done to date. In addition, comparing a group of organic and conventional fruit growers is undoubtedly an enriching aspect of the study. Few studies have examined the relationship between organic food consumption and diet quality, so this study makes a valuable contribution to the state of knowledge on this relationship. Another advantage is that the study uses a 3-day dietary record method. This research, therefore, provides much more precise and comprehensive data than is the case with the conventionally used single 24-h interviews. Finally, the study used two reputable diet-quality indicators, which provided a clear picture of diet quality in the study group. On the other hand, our study also has weaknesses. The relatively small convenience-sampling study group may have distorted the perceived picture of the diet quality of fruit growers. In addition, note-taking took place during one season of the year. A study of the diet in different periods would certainly have given a better picture of the diet quality over the year. Nevertheless, it should be emphasised that the presented research is a pilot study, and further, more extensive studies are needed in this area. It should also be noted that the study used a single-frequency BIA device. Although this is a well-established and effective method in population studies, the use of multi-frequency BIA would certainly provide a more reliable analysis of muscle mass and fat. Finally, it should be emphasised that our study is cross-sectional. This means we used only a single measurement, which cannot provide causal information. However, it provides a lot of important information that can inform the design of other studies, including cohort and experimental studies using organic food to improve diet quality. An additional limitation is that, although total energy intake was available from the dietary records and contributed to the computation of diet-quality indices, it was not included as a separate variable in the present analyses; thus, its possible influence on both diet quality and body composition was not directly assessed. Furthermore, biochemical markers were outside the project’s original scope and were not collected, which limits the interpretation of the clinical and metabolic relevance of the observed differences. Finally, residual confounding related to education, economic circumstances, health consciousness, and other lifestyle characteristics cannot be excluded.

Despite the above limitations, the preliminary results obtained in our study suggest that organic fruit growers have a diet closer to nutritionists’ recommendations than conventional fruit growers, and that organic food consumption is positively correlated with diet quality. This study is a pilot study, however. It therefore indicates some relationships and directions that can guide the planning of further research on a wider group. The present study also suggests the need for nutrition education among fruit growers and farmers in general, as the diet of this professional group appears insufficiently beneficial, especially for conventional producers. These observations should therefore be treated as hypothesis-generating and useful primarily for designing larger studies with broader lifestyle assessment, objective knowledge testing, and, where feasible, biochemical measurements.

## 5. Conclusions

In conclusion, the study group’s diet quality appeared relatively low. Although organic fruit growers tended to exhibit higher diet quality, and diet quality was associated with the proportion of organic food consumed and self-assessed nutritional knowledge, these findings are strictly observational and do not allow for causal inference due to study design, self-reported data, and potential residual confounding. Part of the between-group variation may have reflected unmeasured lifestyle-related characteristics rather than the farming system itself. Therefore, the results should be considered exploratory, and further longitudinal and interventional studies are required to clarify this relationship.

## Figures and Tables

**Table 1:** Demographic characteristics of study participants.

		Organic Group	Conventional Group	*p*-Value *	Overall
Gender	Male	12 22.6%	13 24.5%	0.707	25 47.2%
	Female	12 22.6%	16 30.2%		28 52.8%
Financial situation	Very bad or bad	3 5.7%	1 1.9%	0.496	4 7.5%
	Neither good nor bad	11 20.8%	13 24.5%		24 45.3%
	Good or very good	14 26.4%	11 20.8%		25 47.2%
Education	Secondary or below	11 20.8%	15 28.3%	0.132	26 49.1%
	Higher	17 32.1%	10 18.9%		27 50.9%

* Pearson’s χ^2^ test was used for gender and education. The likelihood ratio was used for the financial situation.

**Table 2:** Characteristics of the study group.

		Age (Years) [Mean ± SD Median Min–Max]	Body Mass (kg) [Mean ± SD Median Min–Max]	Body Height (cm) [Mean ± SD Median Min–Max]	BMI (kg/m^2^) [Mean ± SD Median Min–Max]	PAL [Mean ± SD Median Min–Max]
**Organic**	**Overall**	**46 ± 8** **45** **33–63**	**84 ± 17** **87** **51–113**	**174 ± 8** **172** **161–189**	**28 ± 5** **28** **16–36**	**1.8 ± 0.2** **1.7** **1.4–2.3**
	Orchardist	47 ± 7 47 34–63	90 ± 13 91 65–113	177 ± 7 179 161–189	29 ± 4 28 22–36	1.9 ± 0.3 1.8 1.5–2.3
	Partner	45 ± 9 44 33–62	75 ± 18 70 51–102	170 ± 6 169 161–183	26 ± 6 25 16–36	1.7 ± 0.2 1.7 1.4–2.1
**Conventional**	**Overall**	**43 ± 9** **42** **29–63**	**85 ± 16** **83** **60–113**	**171 ± 10** **169** **154–192**	**29 ± 4** **29** **22–38**	**1.8 ± 0.2** **1.7** **1.5–2.3**
	Orchardist	44 ± 9 44 30–63	91 ± 16 95 64–113	174 ± 12 173 154–192	29 ± 4 29 22–38	1.8 ± 0.2 1.7 1.6–2.3
	Partner	40 ± 8 39 29–57	75 ± 9 75 60–88	166 ± 5 166 161–174	27 ± 3 27 23–34	1.7 ± 0.1 1.7 1.5–1.9
**Overall**	**Overall**	**44 ± 8** **44** **29–63**	**84 ± 16** **86** **51–113**	**172 ± 9** **170** **154–192**	**28 ± 4** **29** **16–38**	**1.77 ± 0.2** **1.7** **1.4–2.3**
	Orchardist	45 ± 8 45 30–63	91 ± 14 95 64–113	176 ± 10 176 154–192	29 ± 4 29 22–38	1.8 ± −0.2 1.8 1.5–2.3
	Partner	43 ± 9 43 29–62	75 ± 15 75 51–102	168 ± 6 167 161–183	26 ± 5 26 16–36	1.7 ± 0.2 1.7 1.4–2.1

**Table 3:** Body composition of study participants.

		Fat Mass (%) [Mean ± SD Median Min–Max]	Fat-Free Mass (kg) [Mean ± SD Median Min–Max]	Total Body Water (%) [Mean ± SD Median Min–Max]
**Organic**	**Overall**	**28.6 ± 7.5** **26.2** **17.4–44.5**	**59.2 ± 11.8** **60.0** **40.0–76.2**	**52.2 ± 5.5** **54.0** **40.7–60.5**
	Orchardist	26.7 ± 6.1 25.9 17.4–41.5	65.8 ± 8.3 67.9 46.9–76.2	53.6 ± 4.5 54.2 42.9–60.5
	Partner	31.2 ± 8.6 29.8 21.2–44.5	50.3 ± 9.8 48.7 40.0–71.6	50.4 ± 6.3 51.5 40.7–57.6
**Conventional**	**Overall**	**31.5 ± 7.4** **31.1** **14.2–45.4**	**57.5 ± 13.1** **57** **.** **0** **41.9 ± 80.2**	**50.2 ± 5.4** **50.4** **40.0–62.8**
	Orchardist	32.5 ± 7.2 32.3 23.5–43.5	57.4 ± 13.3 49.3 43.3–78.9	49.4 ± 5.3 49.6 41.4–56.0
	Partner	30.0 ± 7.8 30.4 14.2–45.4	57.6 ± 13.5 58.3 41.9 ± 80.2	51.3 ± 5.7 51.0 40.0–62.8
**Overall**	**Overall**	**30.0 ± 7.5** **28.5** **14.2 ± 45.4**	**58.4 ± 12.3** **57.8** **40.0–80.2**	**51.3 ± 5.5** **52.4** **40.0–62.8**
	Orchardist	29.5 ± 7.2 26.2 17.4–43.5	61.7 ± 11.6 63.9 43.3–78.9	51.6 ± 5.2 54.0 41.4–60.5
	Partner	30.7 ± 8.1 30.4 14.2–45.4	53.6 ± 11.9 51.2 40.0–80.2	50.8 ± 5.9 51.0 40.0–62.8

**Table 4:** Number of points obtained using the HEI and DQI-I indices.

	HEI [Mean ± SD Median Min–Max]	DQI-I [Mean ± SD Median Min–Max]
**Overall**	**31.7 ± 9.3** **32.3** **16–54**	**54.3 ± 7.5** **54** **33–69**
Conventional	25.8 ± 8.2 23.1 16–54	51.2 ± 6.7 52 33–63
Organic	37.0 ± 6.7 37.0 24–48	57.1 ± 7.1 57 41–69
*p*-value *	**<0.001**	**0.002**

* Student’s *t*-test.

## Data Availability

Dataset available on request from the authors.

## Abbreviations

The following abbreviations are used in this manuscript:

HEI-2015	Healthy Eating Index (2015)
DQI-I	Diet Quality Index-International
EU	European Union
SGGW	Szkoła Główna Gospodarstwa Wiejskiego (ang. Warsaw University of Life Sciences)
NIZP-PZH	Narodowy Instytut Zdrowia Publicznego—Państwowy Zakład Higieny (ang National Institute of Public Health—National Institute of Hygiene
PUFA	Polyunsaturated Fatty Acids
MUFA	Monounsaturated Fatty Acids
SFA	Saturated Fatty Acids
BMI	Body Mass Index
NAFLD	Non-alcoholic Fatty Liver Disease
DQI-V	Diet Quality Index—Vietnamese
BF	Body Fat
FFM	Fat Free Mass
TBW	Total Body Water
